# Bis(2-methyl­quinolin-8-olato-κ^2^ *N*,*O*)­lead(II)

**DOI:** 10.1107/S1600536810012742

**Published:** 2010-04-17

**Authors:** Gholamhossein Mohammadnezhad, Ali Reza Ghanbarpour, Mostafa M. Amini, Seik Weng Ng

**Affiliations:** a Department of Chemistry, General Campus, Shahid Beheshti University, Tehran 1983963113, Iran; b Department of Chemistry, University of Malaya, 50603 Kuala Lumpur, Malaysia

## Abstract

The Pb^II^ atom in the title compound, [Pb(C_10_H_8_NO)_2_], is chelated by two oxine (2-methyl­quinolin-8-olate) anions in a Ψ-trigonal–bipyramidal geometry; the N atoms occupy the axial sites. The mol­ecule lies about a twofold rotation axis.

## Related literature

For the crystal structure of bis­(quinolin-8-olato)lead(II), see: Zhu *et al.* (2005 ▶).
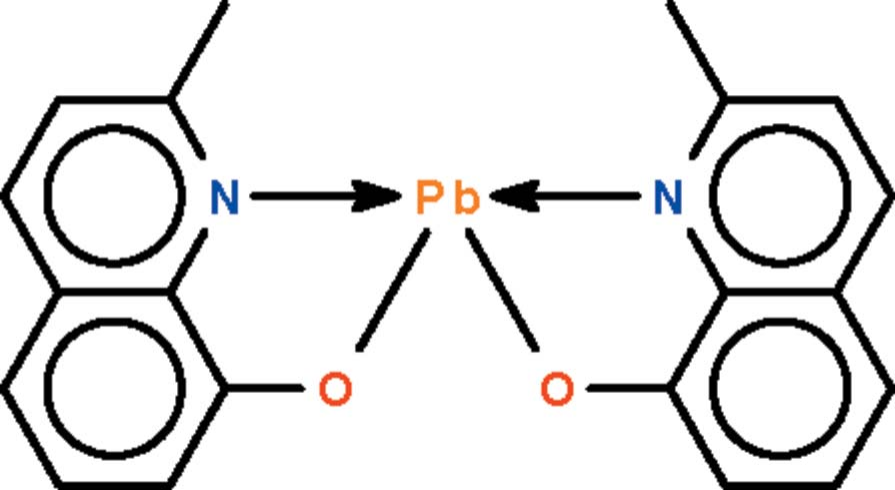


## Experimental

### 

#### Crystal data

[Pb(C_10_H_8_NO)_2_]
*M* *_r_* = 523.54
Monoclinic, 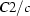

*a* = 22.439 (2) Å
*b* = 4.7636 (5) Å
*c* = 15.7139 (15) Å
β = 101.167 (1)°
*V* = 1647.9 (3) Å^3^
*Z* = 4
Mo *K*α radiation
μ = 10.25 mm^−1^
*T* = 223 K
0.30 × 0.06 × 0.04 mm

#### Data collection

Bruker SMART APEX diffractometer
Absorption correction: multi-scan (*SADABS*; Sheldrick, 1996 ▶) *T*_min_ = 0.149, *T*_max_ = 0.685
7405 measured reflections
1890 independent reflections
1765 reflections with *I* > 2σ(*I*)
*R*_int_ = 0.053

#### Refinement

*R*[*F* ^2^ > 2σ(*F* ^2^)] = 0.027
*wR*(*F* ^2^) = 0.063
*S* = 1.02
1890 reflections
115 parameters
H-atom parameters constrained
Δρ_max_ = 1.69 e Å^−3^
Δρ_min_ = −1.50 e Å^−3^

### 

Data collection: *APEX2* (Bruker, 2009 ▶); cell refinement: *SAINT* (Bruker, 2009 ▶); data reduction: *SAINT*; program(s) used to solve structure: *SHELXS97* (Sheldrick, 2008 ▶); program(s) used to refine structure: *SHELXL97* (Sheldrick, 2008 ▶); molecular graphics: *X-SEED* (Barbour, 2001 ▶); software used to prepare material for publication: *publCIF* (Westrip, 2010 ▶).

## Supplementary Material

Crystal structure: contains datablocks global, I. DOI: 10.1107/S1600536810012742/bt5241sup1.cif

Structure factors: contains datablocks I. DOI: 10.1107/S1600536810012742/bt5241Isup2.hkl

Additional supplementary materials:  crystallographic information; 3D view; checkCIF report

## Figures and Tables

**Table:** 

Pb1—O1	2.262 (3)
Pb1—N1	2.507 (3)

**Table:** 

O1—Pb1—O1^i^	93.6 (2)
N1—Pb1—N1^i^	135.6 (1)

Symmetry code: (i) 
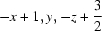
.

**Fig. 1.**
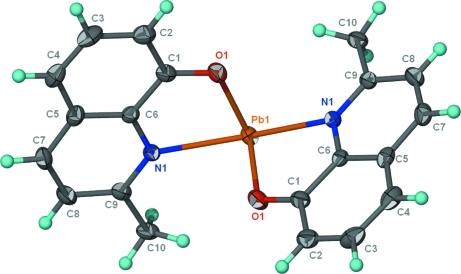
Anisotropic displacement ellipsoid plot (Barbour, 2001) of the title compound; ellipsoids are drawn at the 50% probability level and H atoms are of arbitrary radius.

## supplementary crystallographic information

### Comment

Bis(quinolin-8-olato)lead(II) exists as a centrosymmetric dinuclear entity in which one of the two oxygen atoms also functions as a bridge. As adjacent molecules are linked by a weaker Pb···O interaction to generate a chain motif, the metal atom is regarded as being six-coordinate in a *Ψ*-pentagonal bipyramidal geometry, the lone pair electrons occupying an axial site (Zhu *et al.*, 2005). In the present methyl-substituted analogue, the substituent is able to block the approach of neighboring potentially coordinating atoms so that the compound is only four-coordinate (Scheme I, Fig. 1). The coordination polyhedron is a *Ψ*-trigonal bipyramid and the lone pair electrons occupy an equatorial site. The axial sites are occupied by the nitrogen atoms and the oxygen atoms occupy the other equatorial sites. The lone pair compresses the O–Pb–O angle (Table 1).

### Experimental

Lead (II) acetate trihydrate (1 mmol, 0.38 g), 2-methyl-8-hydroxyquinoline (1 mmol, 0.16 g) and sodium azide (1 mmol, 0.13 g) were loaded in to a convection tube; the tube was filled with 2:1 methanol/water and kept at 333 K. Crystals were collected after 1 week (m.p. > 543 K).

### Refinement

H-atoms were placed in calculated positions (C—H 0.94 Å) and were included in the refinement in the riding model approximation, with *U*(H) set to 1.2*U*(C). The final difference Fourier map had a large peak/deep hole in the vicinity of the lead atom.

### Crystal data

**Table:**

[Pb(C_10_H_8_NO)_2_]	*F*(000) = 992
*M**_r_* = 523.54	*D*_x_ = 2.110 Mg m^−^^3^
Monoclinic, *C*2/*c*	Mo *K*α radiation, λ = 0.71073 Å
Hall symbol: -C 2yc	Cell parameters from 2952 reflections
*a* = 22.439 (2) Å	θ = 2.6–25.2°
*b* = 4.7636 (5) Å	µ = 10.25 mm^−^^1^
*c* = 15.7139 (15) Å	*T* = 223 K
β = 101.167 (1)°	Prism, yellow
*V* = 1647.9 (3) Å^3^	0.30 × 0.06 × 0.04 mm
*Z* = 4	

### Data collection

**Table:**

Bruker SMART APEX diffractometer	1890 independent reflections
Radiation source: fine-focus sealed tube	1765 reflections with *I* > 2σ(*I*)
graphite	*R*_int_ = 0.053
ω scans	θ_max_ = 27.5°, θ_min_ = 1.9°
Absorption correction: multi-scan (*SADABS*; Sheldrick, 1996)	*h* = −28→28
*T*_min_ = 0.149, *T*_max_ = 0.685	*k* = −6→6
7405 measured reflections	*l* = −20→18

### Refinement

**Table:**

Refinement on *F*^2^	Primary atom site location: structure-invariant direct methods
Least-squares matrix: full	Secondary atom site location: difference Fourier map
*R*[*F*^2^ > 2σ(*F*^2^)] = 0.027	Hydrogen site location: inferred from neighbouring sites
*wR*(*F*^2^) = 0.063	H-atom parameters constrained
*S* = 1.02	*w* = 1/[σ^2^(*F*_o_^2^) + (0.030*P*)^2^] where *P* = (*F*_o_^2^ + 2*F*_c_^2^)/3
1890 reflections	(Δ/σ)_max_ = 0.001
115 parameters	Δρ_max_ = 1.69 e Å^−^^3^
0 restraints	Δρ_min_ = −1.50 e Å^−^^3^

### Fractional atomic coordinates and isotropic or equivalent isotropic displacement parameters (Å^2^)

**Table:**

	*x*	*y*	*z*	*U*_iso_*/*U*_eq_	
Pb1	0.5000	0.67661 (5)	0.7500	0.02578 (9)	
O1	0.55706 (16)	0.3516 (7)	0.6978 (2)	0.0336 (8)	
N1	0.57862 (15)	0.4778 (8)	0.8701 (2)	0.0226 (7)	
C1	0.6024 (2)	0.2268 (10)	0.7500 (3)	0.0278 (10)	
C2	0.6392 (2)	0.0290 (11)	0.7206 (3)	0.0344 (11)	
H2	0.6321	−0.0169	0.6613	0.041*	
C3	0.6864 (2)	−0.1029 (11)	0.7771 (4)	0.0393 (13)	
H3	0.7104	−0.2351	0.7547	0.047*	
C4	0.6991 (2)	−0.0466 (10)	0.8640 (4)	0.0355 (11)	
H4	0.7312	−0.1388	0.9008	0.043*	
C5	0.6632 (2)	0.1522 (9)	0.8974 (3)	0.0286 (10)	
C6	0.6149 (2)	0.2861 (9)	0.8407 (3)	0.0237 (9)	
C7	0.6717 (2)	0.2286 (11)	0.9859 (3)	0.0333 (11)	
H7	0.7027	0.1435	1.0266	0.040*	
C8	0.6349 (2)	0.4264 (11)	1.0127 (3)	0.0326 (11)	
H8	0.6414	0.4803	1.0713	0.039*	
C9	0.5878 (2)	0.5476 (10)	0.9526 (3)	0.0263 (9)	
C10	0.5462 (2)	0.7635 (10)	0.9794 (3)	0.0317 (11)	
H10A	0.5048	0.7267	0.9501	0.048*	
H10B	0.5583	0.9490	0.9637	0.048*	
H10C	0.5487	0.7542	1.0417	0.048*	

### Atomic displacement parameters (Å^2^)

**Table:**

	*U*^11^	*U*^22^	*U*^33^	*U*^12^	*U*^13^	*U*^23^
Pb1	0.02798 (15)	0.02687 (14)	0.02138 (14)	0.000	0.00204 (10)	0.000
O1	0.0356 (19)	0.0411 (19)	0.0233 (18)	0.0073 (15)	0.0033 (15)	−0.0031 (14)
N1	0.0225 (18)	0.0247 (18)	0.0207 (18)	−0.0014 (15)	0.0039 (14)	−0.0012 (15)
C1	0.030 (3)	0.030 (2)	0.025 (2)	−0.0004 (19)	0.008 (2)	−0.0019 (19)
C2	0.034 (3)	0.037 (3)	0.034 (3)	−0.002 (2)	0.012 (2)	−0.006 (2)
C3	0.032 (3)	0.034 (3)	0.056 (4)	0.004 (2)	0.019 (3)	−0.006 (2)
C4	0.025 (2)	0.031 (2)	0.050 (3)	0.004 (2)	0.007 (2)	0.004 (2)
C5	0.021 (2)	0.028 (2)	0.035 (3)	−0.0044 (18)	0.003 (2)	0.0039 (19)
C6	0.020 (2)	0.027 (2)	0.025 (2)	−0.0052 (17)	0.0053 (18)	−0.0028 (18)
C7	0.028 (3)	0.039 (3)	0.030 (3)	−0.004 (2)	−0.003 (2)	0.010 (2)
C8	0.036 (3)	0.040 (3)	0.021 (2)	−0.005 (2)	0.004 (2)	0.000 (2)
C9	0.027 (2)	0.028 (2)	0.024 (2)	−0.0103 (19)	0.0062 (18)	−0.0040 (19)
C10	0.036 (3)	0.036 (2)	0.026 (3)	−0.007 (2)	0.012 (2)	−0.008 (2)

### Geometric parameters (Å, °)

**Table:**

Pb1—O1^i^	2.262 (3)	C4—C5	1.409 (7)
Pb1—O1	2.262 (3)	C4—H4	0.9400
Pb1—N1^i^	2.507 (3)	C5—C7	1.414 (8)
Pb1—N1	2.507 (3)	C5—C6	1.416 (7)
O1—C1	1.318 (6)	C7—C8	1.371 (8)
N1—C9	1.316 (5)	C7—H7	0.9400
N1—C6	1.363 (6)	C8—C9	1.399 (7)
C1—C2	1.390 (7)	C8—H8	0.9400
C1—C6	1.426 (7)	C9—C10	1.501 (7)
C2—C3	1.393 (8)	C10—H10A	0.9700
C2—H2	0.9400	C10—H10B	0.9700
C3—C4	1.366 (8)	C10—H10C	0.9700
C3—H3	0.9400		
			
O1—Pb1—O1^i^	93.6 (2)	C4—C5—C7	124.2 (5)
O1^i^—Pb1—N1^i^	69.46 (12)	C4—C5—C6	119.4 (5)
O1—Pb1—N1^i^	80.42 (12)	C7—C5—C6	116.4 (4)
O1^i^—Pb1—N1	80.42 (12)	N1—C6—C5	121.5 (4)
O1—Pb1—N1	69.46 (12)	N1—C6—C1	117.2 (4)
N1—Pb1—N1^i^	135.6 (1)	C5—C6—C1	121.3 (4)
C1—O1—Pb1	120.4 (3)	C8—C7—C5	120.3 (5)
C9—N1—C6	121.0 (4)	C8—C7—H7	119.9
C9—N1—Pb1	126.8 (3)	C5—C7—H7	119.9
C6—N1—Pb1	112.1 (3)	C7—C8—C9	119.9 (4)
O1—C1—C2	122.4 (5)	C7—C8—H8	120.1
O1—C1—C6	120.8 (4)	C9—C8—H8	120.1
C2—C1—C6	116.8 (5)	N1—C9—C8	120.9 (4)
C1—C2—C3	121.5 (5)	N1—C9—C10	117.5 (4)
C1—C2—H2	119.3	C8—C9—C10	121.6 (4)
C3—C2—H2	119.3	C9—C10—H10A	109.5
C4—C3—C2	122.3 (5)	C9—C10—H10B	109.5
C4—C3—H3	118.9	H10A—C10—H10B	109.5
C2—C3—H3	118.9	C9—C10—H10C	109.5
C3—C4—C5	118.8 (5)	H10A—C10—H10C	109.5
C3—C4—H4	120.6	H10B—C10—H10C	109.5
C5—C4—H4	120.6		
			
O1^i^—Pb1—O1—C1	−80.4 (3)	C9—N1—C6—C1	−178.9 (4)
N1^i^—Pb1—O1—C1	−148.9 (4)	Pb1—N1—C6—C1	−1.1 (5)
N1—Pb1—O1—C1	−2.0 (3)	C4—C5—C6—N1	179.4 (4)
O1^i^—Pb1—N1—C9	−83.2 (4)	C7—C5—C6—N1	−0.1 (6)
O1—Pb1—N1—C9	179.2 (4)	C4—C5—C6—C1	−0.9 (7)
N1^i^—Pb1—N1—C9	−130.3 (4)	C7—C5—C6—C1	179.6 (4)
O1^i^—Pb1—N1—C6	99.1 (3)	O1—C1—C6—N1	−0.7 (6)
O1—Pb1—N1—C6	1.6 (3)	C2—C1—C6—N1	−179.4 (4)
N1^i^—Pb1—N1—C6	52.0 (3)	O1—C1—C6—C5	179.6 (4)
Pb1—O1—C1—C2	−179.0 (4)	C2—C1—C6—C5	0.9 (7)
Pb1—O1—C1—C6	2.4 (6)	C4—C5—C7—C8	179.4 (5)
O1—C1—C2—C3	−179.2 (5)	C6—C5—C7—C8	−1.1 (7)
C6—C1—C2—C3	−0.6 (7)	C5—C7—C8—C9	1.7 (7)
C1—C2—C3—C4	0.2 (8)	C6—N1—C9—C8	−0.2 (6)
C2—C3—C4—C5	−0.2 (8)	Pb1—N1—C9—C8	−177.7 (3)
C3—C4—C5—C7	180.0 (5)	C6—N1—C9—C10	179.2 (4)
C3—C4—C5—C6	0.5 (7)	Pb1—N1—C9—C10	1.7 (6)
C9—N1—C6—C5	0.8 (6)	C7—C8—C9—N1	−1.0 (7)
Pb1—N1—C6—C5	178.6 (3)	C7—C8—C9—C10	179.6 (5)

Symmetry codes: (i) −*x*+1, *y*, −*z*+3/2.
